# New aspirin-chitosan conjugates as potential anti-*Staphylococcus aureus* agents

**DOI:** 10.1186/s13065-025-01712-x

**Published:** 2026-01-21

**Authors:** Reham A. Mohamed-Ezzat, Aladdin M. Srour, Sawsan Dacrory

**Affiliations:** 1 Chemistry of Natural and Microbial Products Department, Pharmaceutical and Drug Industries Research Institute, National Research Centre, Dokki, Cairo 12622 Egypt; 2 Department of Therapeutic Chemistry, Pharmaceutical and Drug Industries Research Institute, National Research Centre, Dokki, Cairo 12622 Egypt; 3 Cellulose and Paper Department, National Research Centre, Cairo, Egypt

**Keywords:** Aspirin, Synthesis, Antimicrobial, Staphylococcus aure, Chitosan

## Abstract

Two novel aspirin-chitosan conjugates were successfully designed and synthesized: A phenyl acetate derivative (PAD)/chitosan conjugate and an acetoxybenzoate derivative (ABD)/chitosan conjugate. The conjugates were prepared via freeze-drying methodology, where chitosan was reacted with the phenyl acetate derivative (PAD) **3a** and the acetoxybenzoate derivative (ABD) **3b** through nucleophilic attack of chitosan’s amino groups on the carbonyl carbons of the aspirin-containing derivatives, forming stable amide linkages. The resulting conjugates were comprehensively characterized using infrared spectroscopy (IR), scanning electron microscopy (SEM), and X-ray diffraction (XRD) to confirm successful conjugation and evaluate structural properties. Antibacterial activity was assessed against methicillin-resistant *Staphylococcus aureus* (MRSA) using colony-forming unit (CFU) assays. Results demonstrated that the Cs/3a conjugate exhibited superior antibacterial efficacy, achieving a significant reduction in *S. aureus* growth compared to other formulations. These findings suggest that aspirin-chitosan conjugation represents a promising strategy for developing antimicrobial biomaterials with enhanced therapeutic potential against drug-resistant bacterial pathogens.

**Supplementary Information:**

The online version contains supplementary material available at 10.1186/s13065-025-01712-x.

## Introduction

*Staphylococcus aureus* is one of the main bacterial human pathogens that cause a variety of clinical symptoms [1]. Treatment for both community-acquired and hospital-acquired infections is still challenging due to the emergence of multidrug-resistant strains such as Methicillin-resistant *Staphylococcus aureus* (MRSA) [2, 3]. In addition to being prevalent in the environment and in typical human flora, *S. aureus* is found on the skin and mucous membranes (typically the nasal area) of healthy individuals [1]. On healthy skin, *S. aureus* often does not cause infection, but if it is allowed to penetrate the bloodstream or internal tissues, it can cause many dangerous infections [4, 5]. Infectious endocarditis, bacteremia, skin and soft tissue infections, septic arthritis, osteomyelitis, infections from prosthetic devices, gastroenteritis, pulmonary infections, meningitis, urinary tract infections, and toxic shock syndrome are among the many human infections that are caused by *S. aureus*, one of the most prevalent bacterial infections in humans [6]. Thus, this pathogen causes diseases ranging from simple skin infections to potentially fatal conditions like pneumonia, endocarditis, toxic shock syndrome, and septicemia [7]. Several methicillin-resistant and vancomycin-resistant strains of this pathogen have emerged as a result of antibiotic misuse and overuse. It is noteworthy that all attempts to develop effective staphylococcal vaccinations have thus far been unsuccessful. Thus, the development of small-molecule therapeutic candidates is essential to combating staphylococcal infections that are resistant to antibiotics [8–10]. These challenges underscore the urgent need for alternative therapeutic strategies, including the design of innovative drug delivery systems and antimicrobial biomaterials capable of combating resistant pathogens.

Among natural polymers, chitosan and its derivatives have garnered increasing attention for biomedical use due to their biocompatibility, biodegradability, and antimicrobial activity.

Alkaline deacetylation of chitin, the second most prevalent natural biopolymer on Earth after cellulose, yields chitosan. Chitosan and its derivatives are undisputed biopolymers of significant potential due to their polyelectrolyte features, which include the presence of reactive functional groups, high adsorption capacity, gel-forming ability, complete biodegradability, fungistatic, bacteriostatic, and even anti-tumor properties [11–13]. Many chitosan analogs are also biocompatible and non-toxic to living tissues [14, 15]. Notably, approaches that enable the conjugation of synthetic heterocyclic compounds with biopolymers such as sodium alginate, chitosan, and cellulose are becoming more and more effective. These biopolymers’ special properties, such as their non-toxic nature, cost-effectiveness, biodegradability, and biocompatibility, open up new possibilities for drug manufacturing techniques [12–18]. These biopolymer drug conjugates combine the structural and biological advantages of natural polymers with the pharmacological efficacy of potent compounds, leading to improved solubility, stability, and bioavailability.

It’s worth noting that the chitosan-aspirin combination offers new perspectives into treating virulent and resistant *P. aeruginosa* [18]. Studies have demonstrated that chitosan nanoparticles can modulate quorum-sensing systems, including LasI and RhlI gene expression, thereby reducing virulence factors in clinical isolates of Pseudomonas aeruginosa. Pseudomonas aeruginosa strains that are resistant to antibiotics pose a serious risk for community-acquired infections [19]. Chitosan is a biopolymer with analgesic, antimicrobial, tissue regenerative properties, and biofilm protection [20]. Because of their many uses, antibacterial and biocompatible films have garnered a lot of interest. Research in this sector remains highly active, closely tied to the ongoing development of new materials, despite considerable progress made in this area [21]. Gene therapy, tissue engineering, and cell-based transplantation are crucial therapeutic approaches for regenerative medicine today and in the future. Presenting the target cells in an appropriate matrix to enable their survival during wound contraction, tissue repair, and remodeling in specific tissues is one challenge. 

Chitosan and its derivatives exhibit significant potential as therapeutic agents in regenerative medicine. Their functional properties enable a wide range of applications in tissue repair and regeneration. They can accelerate wound healing by boosting the activity of inflammatory cells and promoting cell repair. Chitosan’s unique characteristics make it suitable for gene therapy, tissue engineering, and cell therapy. Recent research on functional biomaterials has focused on creating better scaffolds and new drug delivery techniques for regenerative medicine. In this context, as part of our ongoing efforts to synthesize bioactive compounds [21–24], we have been working on developing potent inhibitors specifically targeting *S. aureus* [25–28], a major Gram-positive pathogen responsible for many clinical infections. With rising concerns over antibiotic resistance, designing and testing new antimicrobial materials remains critically important.

In this regard, several chitosan-based formulations were synthesized and assessed for their antibacterial efficacy using the CFU-counting method. These composites may have utility in drug delivery systems, offering a promising strategy for combating antibiotic-resistant pathogens.

## Results and discussion

### Chemistry

**Scheme 1:**
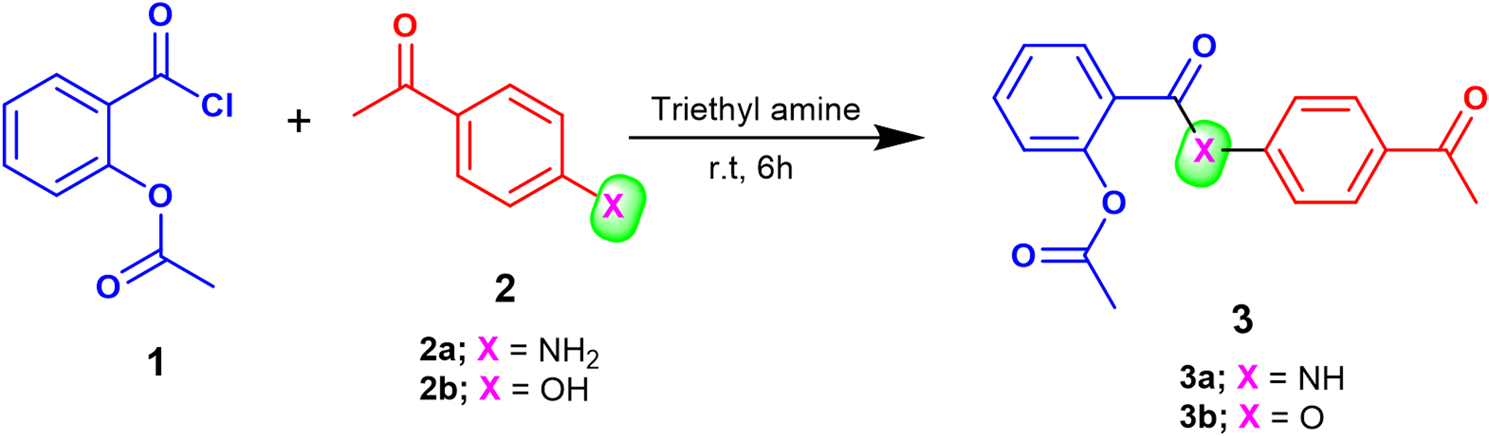
The synthetic route toward the preparation of 2-((4-acetylphenyl)carbamoyl)phenyl acetate (3a) and 4-acetylphenyl 2-acetoxybenzoate (3b)

**Fig. 1:**
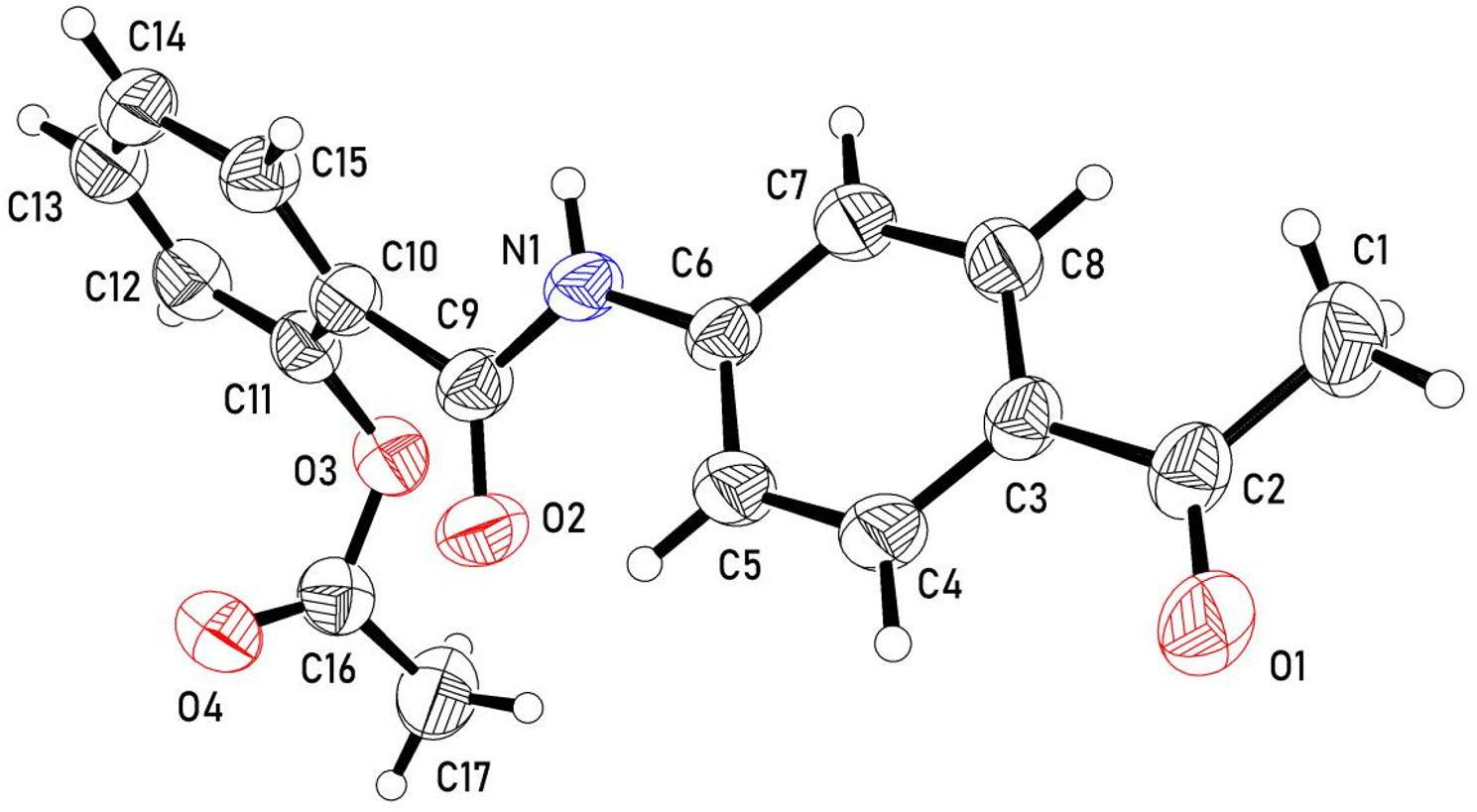
Molecular structure of compound 3a (The International Union of Crystallography has granted permission for the reproduction of the figure depicting the structure through an open-access license) [30].

Two new aspirin-acetophenone chalcone conjugates are designed and synthesized as depicted in Scheme 1. The synthetic path towards synthesizing the desired targets starts from the reaction of the Aspirin’s acid chloride 2-(chlorocarbonyl)phenyl acetate (1) with 4-hydroxy/amino acetophenone (2) to afford the corresponding 2-((4-acetylphenyl)carbamoyl)phenyl acetate (3b) and/or 4-acetylphenyl 2-acetoxybenzoate (3b), respectively. The chemical structure of compounds 3a,b was elucidated using various spectroscopic techniques (Figs S1-S4, supplementary materials). Additionally, Single-Crystal X-ray Diffraction analysis was employed to confirm the structure of compound 3a (Fig. 1) [29–31].

**Fig. 2:**
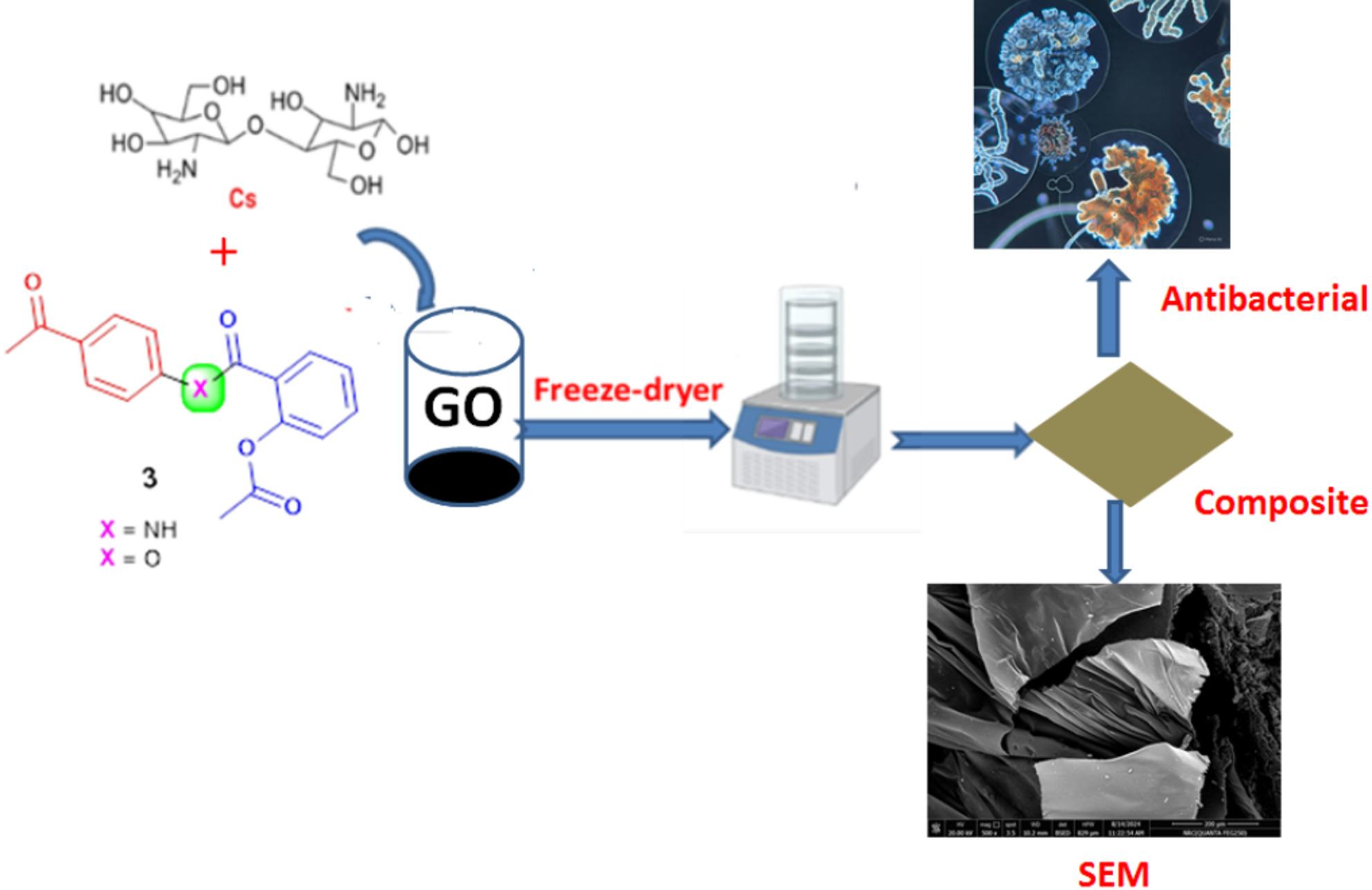
The possible composite preparation

Figure 2 shows the composite preparation based on Cs, GO, phenyl acetate derivative (PAD), and acetoxybenzoate derivative (ABD). Cs reacted via the NH_2_ group with the C = O of PAD and ABD to form a composite, then graphene oxide (GO) is interspersed in pour size of the composite and increases the surface size, which affects the activity of the composite. After the freeze-drying process, which keeps the composite’s fluffy shape.

### FTIR analysis

**Fig. 3:**
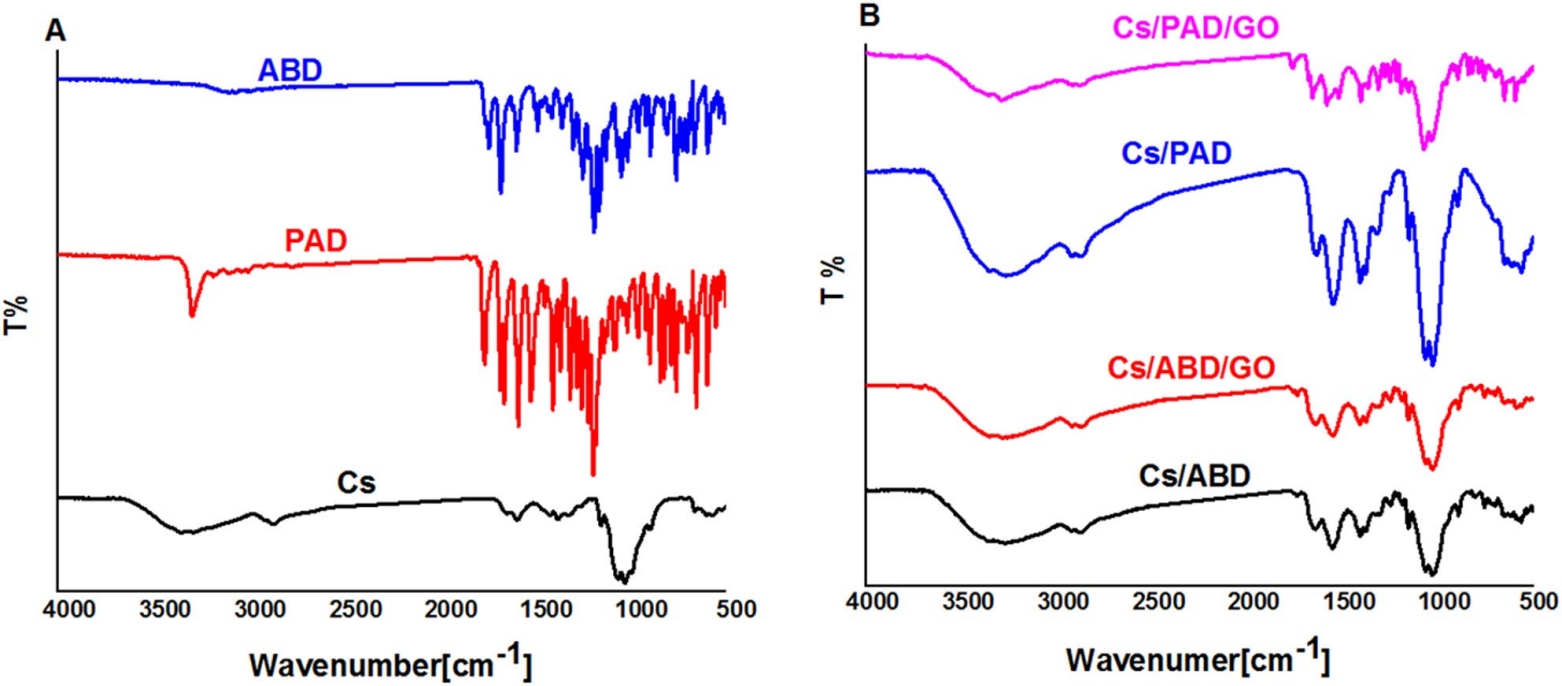
FTIR of Cs, PAD, ABD, and composite

Figure 3 shows the FTIR of raw materials and products. Figure 3A shows the FTIR of Cs and phenyl acetate derivative (PAD) and acetoxybenzoate derivative (ABD). Cs has peaks at 3500 cm^− 1^, 2900 cm^− 1^, 1650 cm^− 1^, and 1100 cm^− 1^ corresponding to overlapping between OH and NH stretching, symmetrical and asymmetric CH vibrations, NH bending, and ether linkage, respectively [32]. The FTIR spectrum of the compound 3a, 2-((4-acetylphenyl)carbamoyl)phenyl acetate (PAD) exhibits several characteristic absorption bands, indicative of key functional groups. A strong and broad band observed at 3297 cm^− 1^ corresponds to the stretching vibration of the NH bond, suggesting the presence of an amide or related NH-containing group. The spectrum also shows prominent carbonyl (C = O) stretching bands at 1659 cm^− 1^, 1679 cm^− 1^, and 1760 cm^− 1^, which are consistent with the presence of multiple carbonyl functionalities. Specifically, the band at 1760 cm^− 1^ is characteristic of an ester carbonyl, while the absorptions at 1659 cm^− 1^ and 1679 cm^− 1^ are typical of the amide group, possibly indicating conjugation or hydrogen bonding effects. These IR features support the presence of amide and ester groups within the molecular structure.

The IR spectrum of the compound 3b, 4-acetylphenyl 2-acetoxybenzoate (ABD) reveals several distinct carbonyl stretching vibrations, indicating the presence of multiple carbonyl-containing functional groups. Absorption bands at 1656 cm^− 1^ and 1682 cm^− 1^ are characteristic of amide carbonyl groups, respectively. These peaks suggest involvement in resonance or hydrogen bonding interactions. Additionally, a sharp and intense band at 1756 cm^− 1^ is typical of an ester carbonyl, reflecting the presence of a more electron-deficient and less conjugated carbonyl group. Collectively, these IR signals confirm the coexistence of amide and ester functionalities in the compound (Figs S5-S8, supplementary materials).

Figure 3B shows the FTIR of products, Cs and GO with phenyl acetate derivative (PAD) and acetoxybenzoate derivative (ABD). A new peak was detected in the Cs chain at 1700 cm^− 1^ due to the Schiff base’ C = N group, demonstrating that the present functional groups became more clearly [33].

### Scanning electron microscope (SEM)

**Fig. 4:**
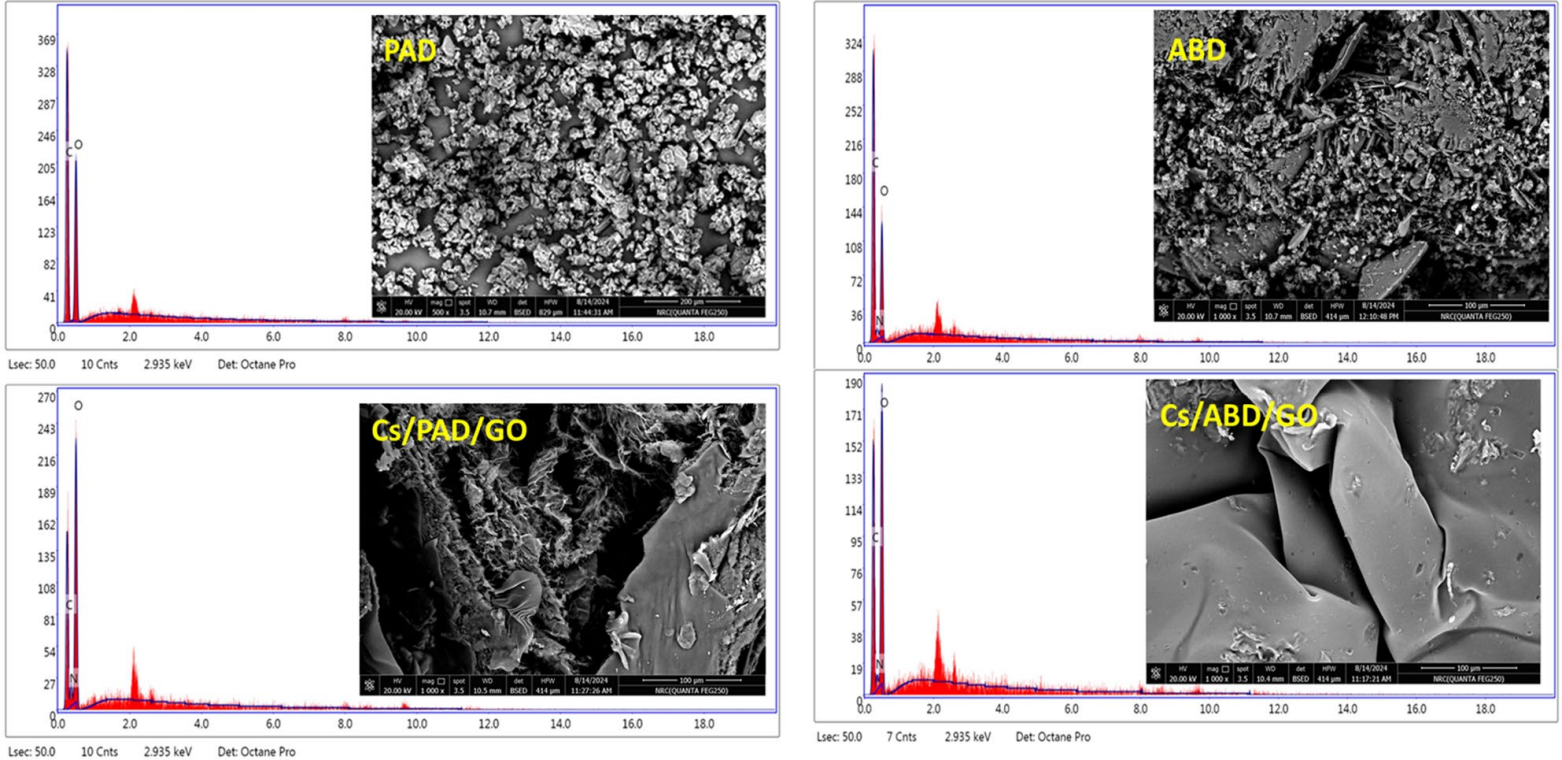
SEM of PAD, ABD, and composite

Figure 4 illustrates the surface morphology of PAD, ABD, and their composite, as analyzed using scanning electron microscopy. The surfaces of PAD and ABD exhibit a dispersed arrangement of light particles. After undergoing the Cs reaction and subsequent freeze-drying process, the morphology evolved into distinct layered structures. Furthermore, energy-dispersive X-ray (EDX) images confirm the presence of key elements-oxygen (O), carbon (C), and nitrogen (N) in both the composite and ABD, indicating their integral role in the material’s composition.

### X-ray diffraction

**Fig. 5:**
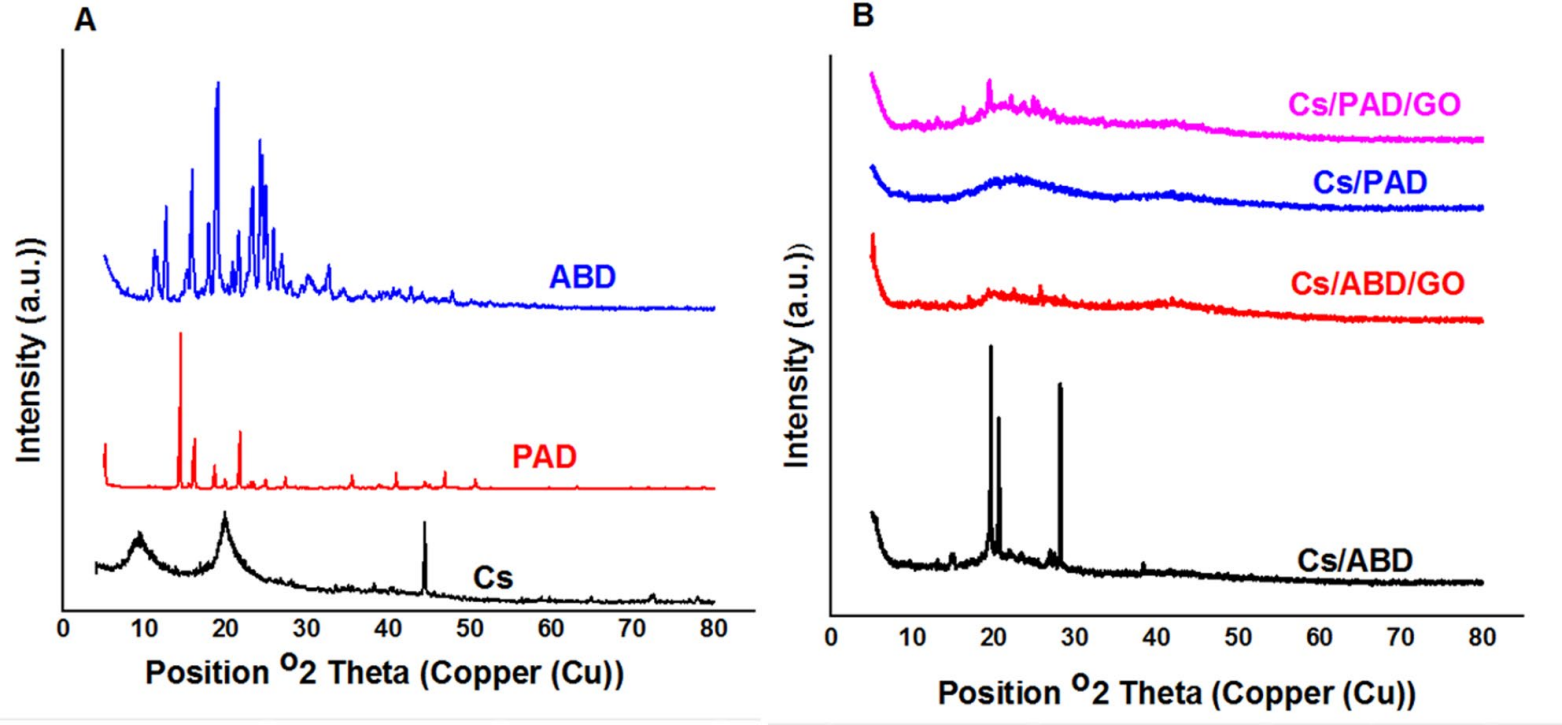
XRD of Cs, PAD, ABD, and composite

Figure 5 illustrates the XRD patterns of the raw materials (Cs, PAD, ABD) as well as the product composites containing Cs and GO. The XRD pattern of Cs reveals characteristic peaks at 2θ = 10° and 20°, corresponding to the amorphous and crystalline regions, respectively. In contrast, PAD and ABD exhibit sharp peaks indicative of higher crystallinity. However, these peaks appear diminished and broadened in the composites, suggesting enhanced homogeneity in the resulting materials, (Figs S9-S12, supplementary materials).

### Antimicrobial evaluation

The antibacterial activity of compounds 3a and 3b, along with their polymer-based formulations, Cs/3a, Cs/3b, Cs/3a/GO, and Cs/3b/GO, was evaluated against *Staphylococcus aureus* using a colony-forming unit (CFU) assay, with results compared to a control. The CFU counts and calculated antibacterial activity percentages (% inhibition) reveal clear trends regarding the efficacy of each compound. The results obtained revealed a clear hierarchy of antimicrobial effectiveness, with pure compounds 3a and 3b demonstrating exceptional activity (93.8% and 96.9% inhibition, respectively) and significant log reductions that would be considered clinically meaningful. However, incorporation into chitosan matrices resulted in substantially reduced activity, particularly for compound 3b (from 96.9% to 15.6% inhibition), while compound 3a maintained relatively high efficacy (89.1%) in the chitosan composite, suggesting differential compatibility with the polymer matrix. The addition of graphene oxide (GO) further compromised antimicrobial performance, with both Cs/3a/GO and Cs/3b/GO exhibiting poor activity (15.6% and 20.3% respectively) and CFU counts approaching control levels, indicating that GO incorporation may interfere with antimicrobial mechanisms through physical entrapment of active compounds, chemical interactions that reduce bioactivity, altered surface morphology preventing bacterial contact, or aggregation effects creating heterogeneous distribution of active sites. While the pure compounds demonstrate promising antimicrobial potential, the dramatic activity loss upon composite formation highlights the need for formulation optimization to balance the enhanced material properties provided by Cs and GO incorporation against the preservation of therapeutic efficacy, requiring future investigation of controlled release mechanisms, loading ratios, and alternative composite architectures. The negative control served as the baseline for bacterial growth, exhibiting maximal proliferation at 2.4 × 10^6^ CFU/mL, indicative of unrestrained bacterial multiplication under the given experimental conditions.

**Table:** 

*S. aureus*	3a	3b	Cs/3a	Cs/3b	Cs/3a/GO	Cs/3b/GO	Control
Dilution Factor	10^-3^	10^-3^	10^-3^	10^-3^	10^-3^	10^-3^	10^-3^
Volume of broth plated (*µ*L)	20 *µ*L	20 *µL*	20 *µL*	20 *µL*	20 µL	20 *µL*	20 *µL*
CFU at the dilution factor	4	60	2	7	54	51	64
Total CFU/mL	150000	2250000	75000	262500	2025000	1912500	2400000
Log total CFU	5.18	6.35	4.88	5.42	6.31	6.28	6.38
%	93.8%	6.3%	96.9%	89.1%	15.6%	20.3%	-

Colony-forming unit (CFU)

The percent of inhibition is calculated according to the following equation: PI = (CFU of Control - CFU of Drug) / CFU of Control * 100

## Materials and methods

Melting points were determined on a Stuart SMP30 melting-point apparatus. Infrared (IR) spectra (KBr) were obtained using a JASCO 6100 spectrophotometer. NMR Spectra were recorded on a JEOL AS 500 spectrometer (JEOL USA, Inc.) in DMSO-d_6_ at 500 MHz. Mass spectra were obtained on a Shimadzu GC-MS-QP 1000 EX mass spectrometer (EI, 70 eV), Shimadzu Corporation, Kyoto, Japan. Elemental analyses were performed by a Vario Elemental Analyzer. Chitosan (Cs) with a deacetylation degree of > 90% was purchased from Sigma Aldrich. Graphene oxide (GO) was prepared and characterized according to previous work [34]. Starting material, 2-(chlorocarbonyl)phenyl acetate (1), was prepared according to a literature method [35, 36].

### Chemistry

#### General procedure for the synthesis of 2-((4-acetylphenyl)carbamoyl)phenyl acetate (3a) and 4-acetylphenyl 2-acetoxybenzoate (3b)

To a stirred mixture of *N*,* N*-diethylethanamine (1.48 mL, 11 mmol) and either 4`-aminoacetophenone (1a) or 4`-hydroxyacetophenone (1b) (10 mmol) in THF 25 mL, 2-(chlorocarbonyl)phenyl acetate (2) (1.98 gm, 10 mmol) was added gradually while maintaining the temperature at 0 °C. After the addition was complete, the mixture was allowed to stir at room temperature (25–30 °C) for 16 h. The resulting mixture was then poured into ice-cold water, and the formed solid was collected, washed, dried, and recrystallized from benzene/pet. ether 60–80 and Ethyl acetate, respectively, to obtain the corresponding desired products (3a,b).

##### 2-((4-Acetylphenyl)carbamoyl)phenyl acetate (3a)

Buff crystals; yield (2.65 gm) 89%; mp 151–153 °C; IR (*ν*_max_/cm^− 1^): 3297 (NH), 1659,1679, 1760 (C = O); ^1^H NMR (DMSO-d_6_) *δ* (ppm): 2.18 (s, 3 H, CH_3_), 2.52 (s, 3 H, COCH_3_), 7.24, 7.26 (dd, 1H, *J* = 1.20, 1.10 Hz, CH), 7.39 (t, 1H, *J* = 8.13 Hz, CH), 7.57 (t, 1H, *J* = 8.65 Hz, CH), 7.70, 7.71 (dd, 1H, *J* = 1.65, 1.65 Hz, CH), 7.85 (d, 2 H, *J* = 8.8 Hz, CH), 7.94 (d, 2 H, *J* = 8.8 Hz, CH), 10.71 (s, 1H, NH); ^13^C NMR (DMSO-d_6_) *δ* (ppm) 20.84, 26.60, 119.28, 123.48, 126.08, 129.37, 129.45, 129.53, 129.57, 132.03, 132.32, 143.64, 148.30, 164.80, 169.04, 196.74; MS: m/z (%) 297.88 (M^+^, 9.97); Anal. calcd. for C_17_H_15_NO_4_ (297.31): C, 68.68; H, 5.09; N, 4.71. Found: C, 68.66; H, 5.10; N, 4.70.

##### 4-Acetylphenyl 2-acetoxybenzoate (3b)

Tan brown micro crystals; yield (2.43 gm) 81.5%; mp 143–145 °C; IR (*ν*_max_/cm^− 1^): 1656, 1682, 1756 (C = O); ^1^H NMR (DMSO-d_6_) *δ* (ppm): 2.29 (s, 3 H, COCH_3_), 2.59 (s, 3 H, COCH_3_), 7.16 (d, 1H, *J* = 6.9 Hz, CH), 7.38 (t, 2 H, *J* = 8.8 Hz, CH), 7.38 (t, 1H, *J* = 7.0 Hz, CH), 7.64 (t, 1H, *J* = 7.0 Hz, CH), 8.02 (d, 2 H, *J* = 8.8 Hz, CH), 8.20 (d, 1H, *J* = 6.2 Hz, CH); ^13^C NMR (DMSO-d_6_) *δ* (ppm) 21.01, 26.62, 58.36, 122.02, 122.22, 124.19, 126.32, 130.14, 132.23, 134.99, 135.09, 151.38, 154.35, 162.47, 169.69, 196.90; MS: m/z (%) 298.55 (M^+^, 15.28); Anal. calcd. for C_17_H_14_O_5_ (298.29): C, 68.45; H, 4.73. Found: C, 68.39; H, 4.76.

### Characterizations

The infrared spectra (FTIR) were obtained in the region 4000–400 cm^−1^ using a Shimadzu 8400 S FTIR spectrophotometer. Morphology of the surface was studied using a FEI Inspect S scanning electron microscope (Philips, Poland) in environmental mode without coating; the results were then analyzed with a JEOL JEM-2100 electron microscope at 100,000× with an acceleration voltage of 120 kV. X-ray diffraction (XRD) patterns were recorded from the Diano X-ray diffractometer using a CuK*α* radiation source.

### Preparation of the composites

A homogeneous solution of Cs was prepared via dissolving 5 g of Cs in 100 mL of distilled H_2_O in the presence of 2% acetic acid with continuous stirring. 10 mL of Cs was mixed with GO and aspirin-containing derivative [(2-[(4-acetylphenyl)carbamoyl]phenyl acetate (PAD) and acetoxybenzoate (ABD)] individually, as in the table. The mixture was allowed to stir for 30 min. at room temperature, then it was poured into a petri dish and frozen at −20 °C, followed by a lyophilization process [37] (Table 1).

**Table 1:** The chemical composition of the prepared composite

Sample	Cs(g)	PAD(g)	ABD(g)	GO(g)
Cs/ABD	0.5	-	0.1	-
Cs/ABD/GO	0.5		0.1	0.05
Cs/PAD	0.5	0.1	-	-
Cs/PAD/GO	0.5	0.1		0.05

### Antimicrobial activity

#### Colony forming unit counting test (CFU)

*Staphylococcus aureus* bacteria were used to check how well the polymer sheets fight germs, and the team worked with the usual agar plate method. First, a Staph suspension matching the McFarland 0.5 standard was made and grown in Mueller-Hinton broth so its density stayed steady. Next, 200 *µ*L of that broth was pipetted into a 96-well plate already filled with test samples and a DMSO control. The whole plate sat at 37 °C for 24 h so the polymer and bacteria could interact. After that, 20 *µ*L was taken from each well and spread on dry nutrient agar to see how many cells survived. Those new plates were again held at 37 °C for a full day until clear colonies formed. A digital camera then snapped photos of the plates, and a simple count gave the final colony numbers. From these counts, the researchers calculated antibacterial efficacy as outlined [38].

$$ \begin{array}{*{20}c} {{\mathrm{Antibacterial}}} \\ {{\mathrm{ratio}}(\% )} \\ \end{array} {\text{ }} = \frac{{\left( \begin{gathered} \begin{array}{*{20}c} {{\text{Number of CFUs}}} \\ {{\text{in control group}}} \\ \end{array} {\text{ }} \hfill \\ {\text{ }} - {\text{ }}\begin{array}{*{20}c} {{\text{Number of CFUs}}} \\ {{\text{in experimental group}}} \\ \end{array} {\text{ }} \hfill \\ \end{gathered} \right){\text{ }} \times {\mathrm{1}}00\% }}{{\begin{array}{*{20}c} {{\text{Number of CFUs }}} \\ {{\text{in the control group}}} \\ \end{array} ~}} $$

## Conclusion

The successful synthesis and characterization of new aspirin-chitosan conjugates, especially the Cs/3a (PAD) derivative, highlight their potential as effective antibacterial agents. The enhanced inhibition of methicillin-resistant *Staphylococcus aureus* (MRSA) growth by Cs/3a emphasizes its promise as a basis for developing antimicrobial biomaterials. These findings support the viability of aspirin-chitosan conjugation as an innovative approach to combat drug-resistant bacterial infections and open avenues for future therapeutic applications.

## Supplementary Information

Supplementary Material 1.

## Data Availability

The datasets used and/or analysed during the current study are available from the corresponding author on reasonable request.

## Abbreviations

ABD: Acetoxybenzoate derivative
CFU: Colony-Forming Unit
Cs: Chitosan
EDX: Energy-dispersive X-ray
GO: Graphene oxide
MRSA: Methicillin-Resistant *Staphylococcus aureus*
*S. aureus*: Staphylococcus aureus
SEM: Scanning Electron Microscopy
IR: Infrared Spectroscopy
*P. aeruginosa*: *Pseudomonas aeruginosa*
PAD: Phenyl acetate derivative
XRD: X-ray Diffraction
