# Supplementary material for: New aspirin-chitosan conjugates as potential anti-Staphylococcus aureus agents
Source: BMC Chem. 2026 Jan 21;20(1):28. doi: 10.1186/s13065-025-01712-x (PMC12905951; doi:10.1186/s13065-025-01712-x)
Supplement: Supplementary file 1 — Supplementary Material 1. [file 13065_2025_1712_MOESM1_ESM.docx]

**Supplementary file**

New Aspirin-Chitosan Conjugates as Potential Anti-*Staphylococcus Aureus* Agents

Reham A. Mohamed-Ezzat^a^, [Aladdin M Srour](https://pubmed.ncbi.nlm.nih.gov/?term=Srour+AM&cauthor_id=34775204)^b*^, Sawsan Dacrory^c^

*^a^*Chemistry of Natural and Microbial Products Department, Pharmaceutical and Drug Industries Research Institute, National Research Centre, Dokki, Cairo 12622, Egypt.

*^b^*Department of Therapeutic Chemistry, Pharmaceutical and Drug Industries Research Institute, National Research Centre, Dokki, Cairo 12622, Egypt.

^c^Cellulose and Paper Department, National Research Centre, Cairo, Egypt.

Corresponding author: [am.srour@nrc.sci.eg](mailto:am.srour@nrc.sci.eg)

**Fig. S1.** ^1^H NMR spectrum of compound **3a**.

**Fig. S2.** ^13^C NMR spectrum of compound **3a**.

**Fig. S3.** ^1^H NMR spectrum of compound **3b**.

**Fig. S4.** ^13^C NMR spectrum of compound **3b**.


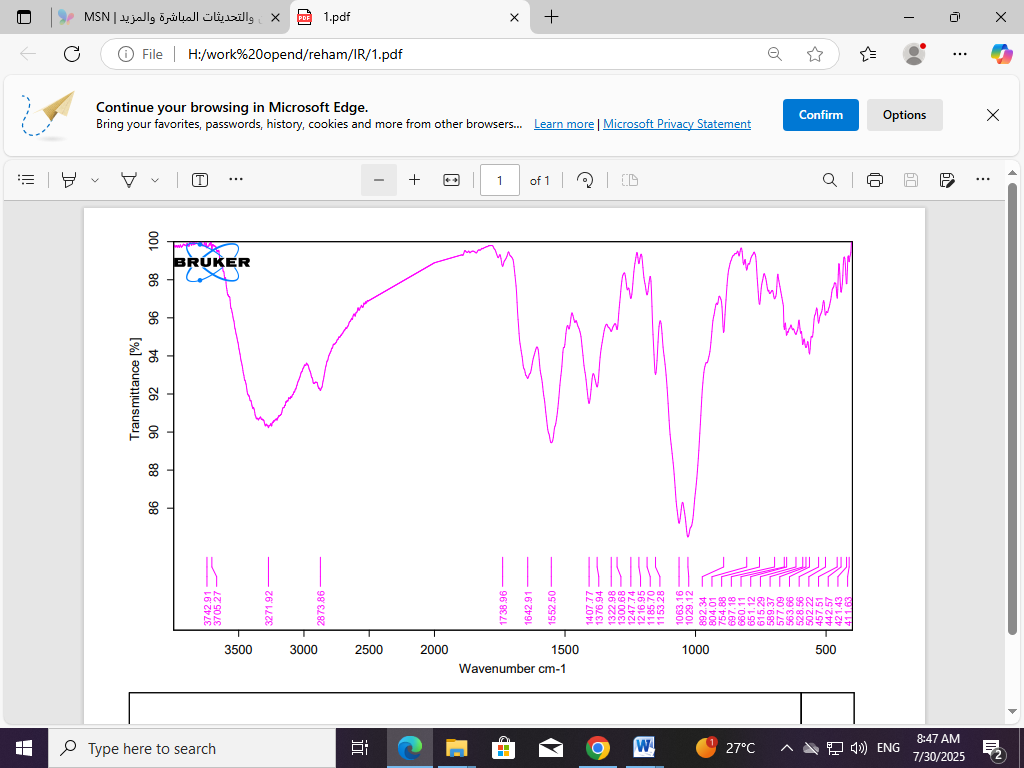


**Fig. S5**. IR Spectrum of Cs/ABD


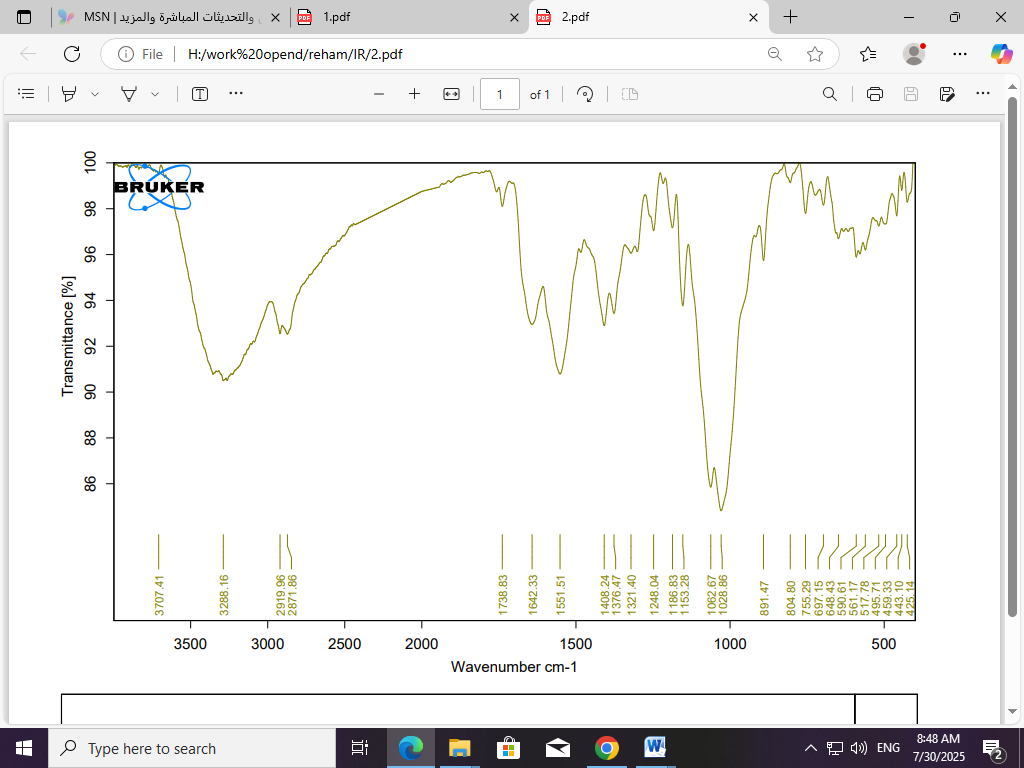


**Fig. S6**. IR Spectrum of Cs/ABD/GO


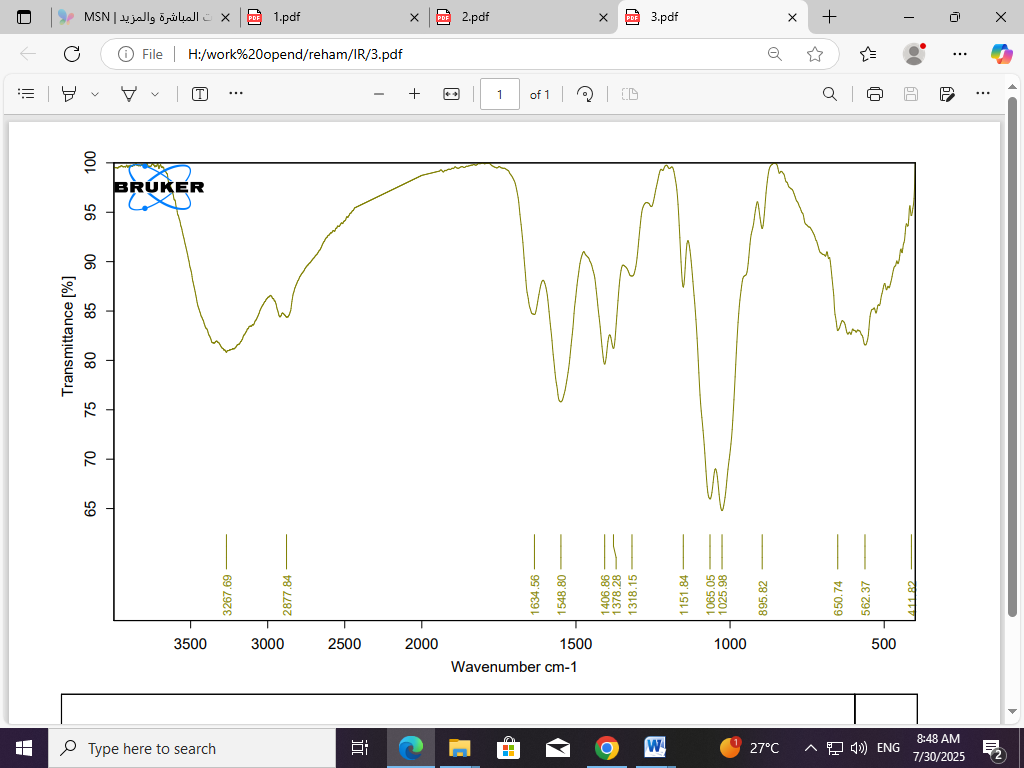


**Fig. S7**. IR Spectrum of Cs/PAD


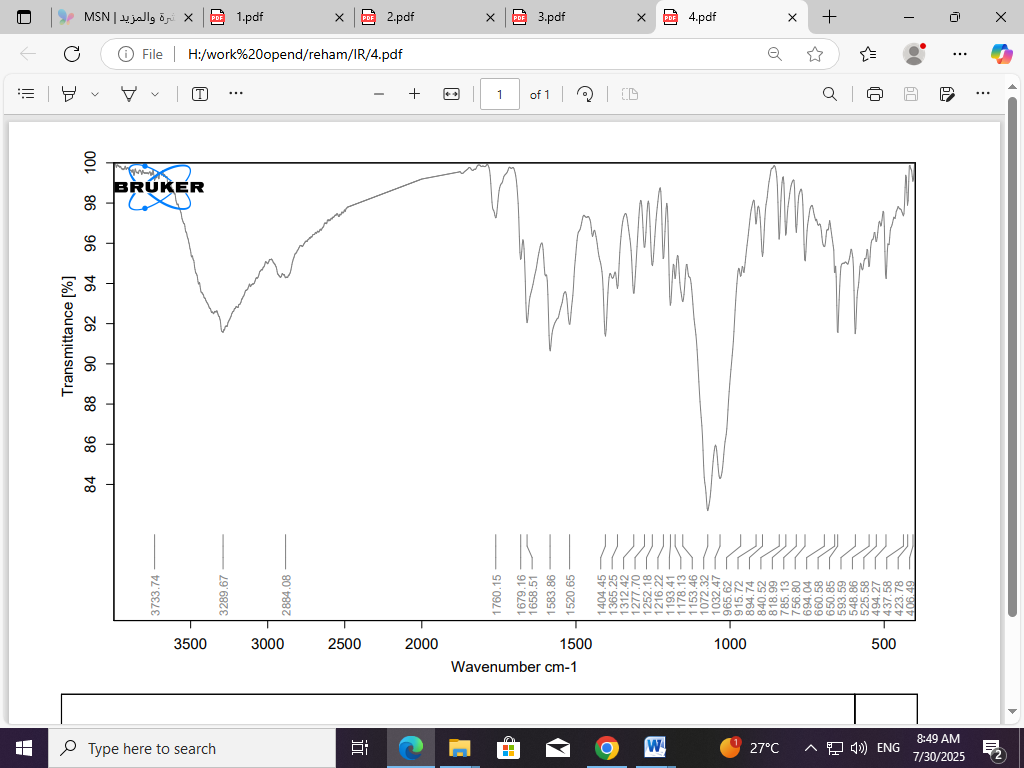


**Fig. S8**. IR Spectrum of Cs/PAD/GO


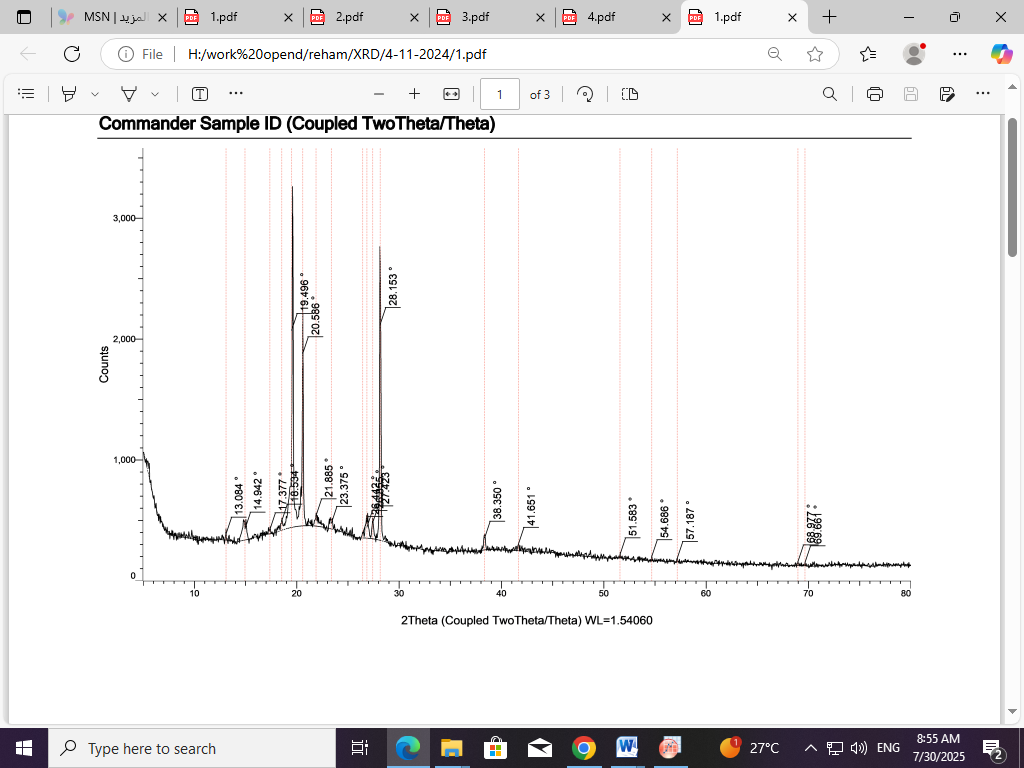


**Fig. S9**. XRD Spectrum of Cs/ABD


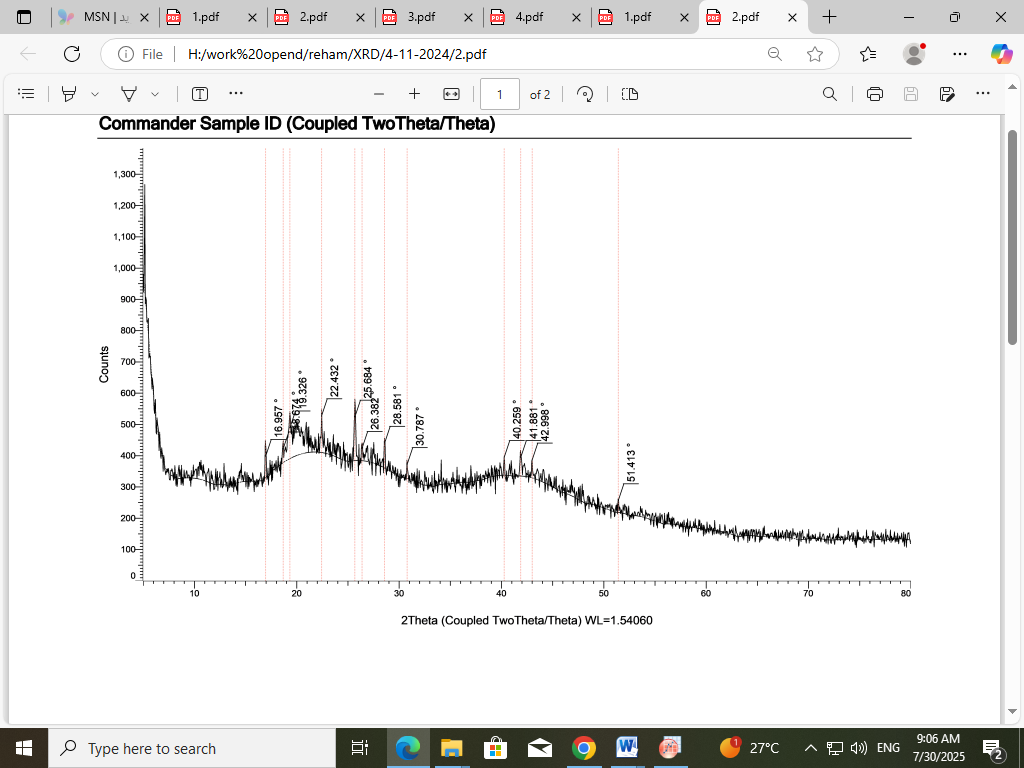


**Fig. S10**. XRD Spectrum of Cs/ABD/GO


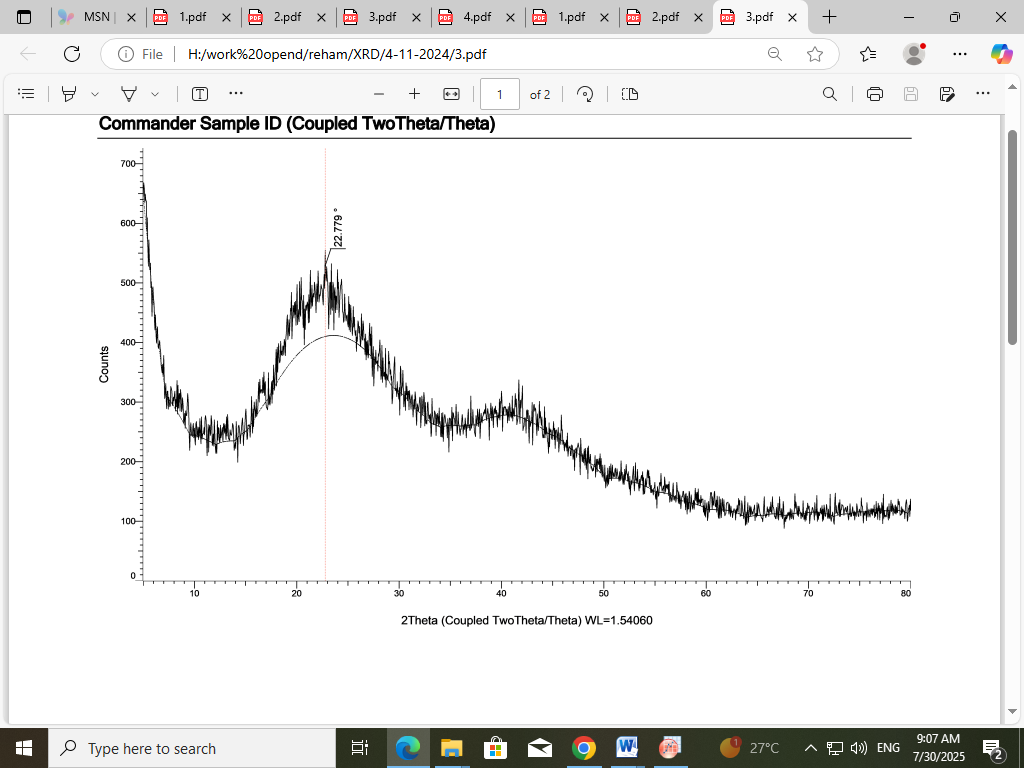


**Fig. S11**. XRD Spectrum of Cs/PAD


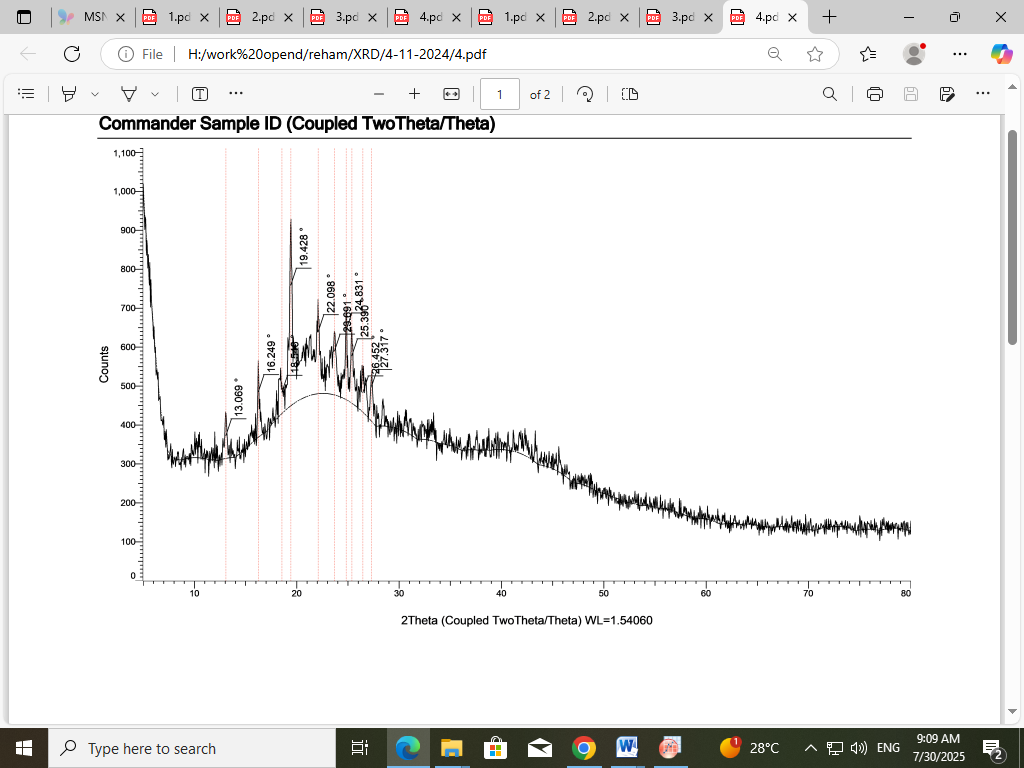


**Fig. S12**. XRD Spectrum of Cs/PAD/GO
